# Transcranial Magnetic Stimulation Improves Muscle Involvement in Experimental Autoimmune Encephalomyelitis

**DOI:** 10.3390/ijms22168589

**Published:** 2021-08-10

**Authors:** Maria Angeles Peña-Toledo, Evelio Luque, Ignacio Ruz-Caracuel, Eduardo Agüera, Ignacio Jimena, Jose Peña-Amaro, Isaac Tunez

**Affiliations:** 1 Dementia and Multiple Sclerosis Unit, Neurology Service, Reina Sofia University Hospital, 14004 Cordoba, Spain; angelespe87@gmail.com (M.A.P.-T.); doctoredu@gmail.com (E.A.); 2 Maimonides Institute for Biomedical Research IMIBIC, Reina Sofia University Hospital, University of Cordoba, 14004 Cordoba, Spain; cm1lucae@uco.es (E.L.); cm1jimei@uco.es (I.J.); 3 Department of Morphological Sciences, Section of Histology, Faculty of Medicine and Nursing, University of Cordoba, 14004 Cordoba, Spain; iruzcaracuel@gmail.com; 4 Department of Biochemistry and Molecular Biology, Faculty of Medicine and Nursing, University of Cordoba, 14004 Cordoba, Spain; 5 Cooperative Research Thematic Excellent Network on Brain Stimulation (REDESTIM), Ministery for Economy, Industry and Competitiveness, 28046 Madrid, Spain

**Keywords:** experimental autoimmune encephalomyelitis, multiple sclerosis, skeletal muscle, transcranial magnetic stimulation, natalizumab, oxidative stress

## Abstract

Skeletal muscle is affected in experimental autoimmune encephalomyelitis (EAE), which is a model of multiple sclerosis that produces changes including muscle atrophy; histological features of neurogenic involvement, and increased oxidative stress. In this study, we aimed to evaluate the therapeutic effects of transcranial magnetic stimulation (TMS) on the involvement of rat skeletal muscle and to compare them with those produced by natalizumab (NTZ). EAE was induced by injecting myelin oligodendrocyte glycoprotein (MOG) into Dark Agouti rats. Both treatments, NTZ and TMS, were implemented from day 15 to day 35. Clinical severity was studied, and after sacrifice, the soleus and extensor digitorum longus muscles were extracted for subsequent histological and biochemical analysis. The treatment with TMS and NTZ had a beneficial effect on muscle involvement in the EAE model. There was a clinical improvement in functional motor deficits, atrophy was attenuated, neurogenic muscle lesions were reduced, and the level of oxidative stress biomarkers was lower in both treatment groups. Compared to NTZ, the best response was obtained with TMS for all the parameters analyzed. The myoprotective effect of TMS was higher than that of NTZ. Thus, the use of TMS may be an effective strategy to reduce muscle involvement in multiple sclerosis.

## 1. Introduction

Experimental autoimmune encephalomyelitis (EAE) causes neuroinflammatory lesions similar to those seen in multiple sclerosis (MS), making it a useful model to test different therapeutic strategies [1]. Our group has previously shown that the use of transcranial magnetic stimulation (TMS) has neuroprotective effects in the EAE model. TMS reduces the proliferation of astrocytes and the number of pyknotic neurons, together with a reduction of oxidative stress parameters [2,3]. A similar neuroprotective effect was also previously demonstrated in a study using an experimental model of Huntington disease [4].

Skeletal muscle motor capacity is impaired in patients with neurodegenerative diseases as a result of being a target nervous system organ [5]. Thus, in MS, muscle function is impaired, and patients show significant motor weakness, leading to substantial disability [6,7,8,9]. Muscle biopsies both from patients with MS [10,11] and from animal EAE models [12] usually show muscle fiber atrophy and fiber-type changes. In a previous study, we showed that skeletal muscle is severely affected in the EAE model, showing an evident histological pattern of neurogenic lesions containing atrophic and angulated muscle fibers. Moreover, there were cytoarchitectural lesions (target, core-targetoid, and moth-eaten fibers) associated to increased expression of oxidative stress biomarkers [13]. Given that TMS effectively attenuates motor impairment secondary to EAE, resulting in clinical improvement in terms of mobility [2,3], we believe that it is also important to understand the potential effects of this treatment on muscle in this experimental model.

The histological changes caused by TMS in skeletal muscle have been little studied, although it has shown beneficial effects in experimental models of post-injury regeneration. We previously demonstrated the protective effect of TMS in a myotoxic muscle damage model, which is characterized by the restoration of oxidative stress biomarkers, increased levels of nitric oxide, and an increase in the number and size of regenerative muscle fibers [14]. Moreover, in an experimental model of contusion injury, Stölting et al. [15] showed that magnetic stimulation favored muscle and nerve regeneration by activating the interaction between muscles and nerves and by inducing the maturation of neuromuscular junctions. Furthermore, when used as a method to induce muscle activity, magnetic stimulation has been shown to delay the muscle atrophy caused by denervation [16]. Indeed, it was recently reported that neuromuscular magnetic stimulation produces a clinical improvement in muscle strength by counteracting the muscle atrophy present in amyotrophic lateral sclerosis [17] and in the paretic limb atrophy in patients with cerebrovascular accident [18]. Furthermore, lumbar spinal root magnetic stimulation also prevented fibrosis in rats immobilized in a shortened muscle position [19].

All the above suggests that TMS could have a myoprotective effect in patients with MS. To verify this hypothesis, we analyzed the effects of TMS on muscle involvement in the EAE model in terms of histological morphology and the expression of oxidative stress markers. For comparative purposes, we also analyzed the effects of natalizumab (NTZ) in this model as one of the current treatments for MS [20]. To the best of our knowledge, this is the first time the effects of TMS and NTZ on skeletal muscle in the EAE model have been studied.

## 2. Results

### 2.1. Mobility Scale

**Figure 1:**
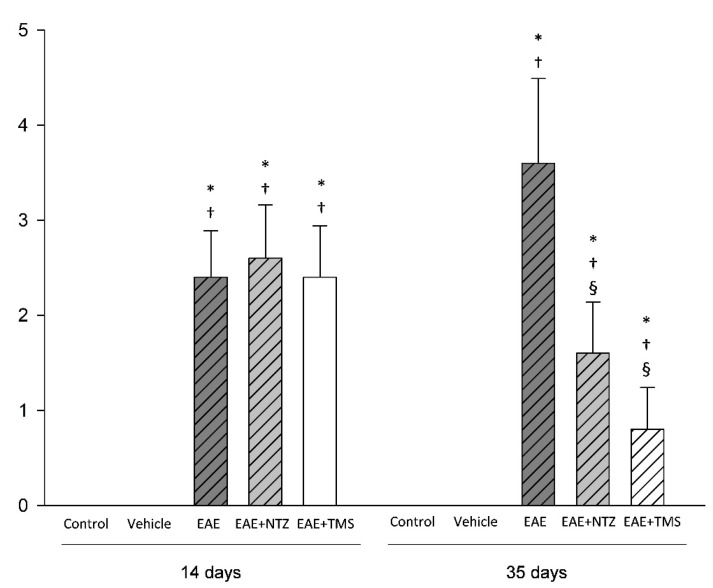
Clinical rating at the beginning (day 14) and at the end of treatments (day 35). All the values are expressed as the mean ± standard deviation (SD). * *p* < 0.05 versus the control group; ^†^ *p* < 0.05 versus the vehicle group; ^§^ *p* < 0.05 versus the experimental autoimmune encephalomyelitis (EAE) group.

A mobility scale was used to clinically measure the course of the disease. Control and vehicle groups had scores of 0, corresponding to healthy animals during the whole experiment. At day 14, when treatments were started (Supplementary Figure S1), there were no significant differences among the three groups that developed the disease. Mean mobility scale scores were 2.4, 2.6, and 2.4 in the EAE, EAE + NTZ, and EAE + TMS groups, respectively (Figure 1). In contrast, after three weeks of treatment initiation (day 35), animals in the EAE group had clinically worsened with a mean score of 3.6, while animals in the EAE + NTZ and EAE + TMS groups had clinically improved with a mean score of 1.7 and 0.9, respectively (Figure 1).

### 2.2. Biochemistry

To assess muscle oxidative stress, a representative white (soleus) and red (extensor digitorum longus—EDL) were selected. The glutathione system was measured by the reduced/oxidized glutathione ratio (GSH/GSSG). Products of oxidative stress such as carbonylated proteins and lipoperoxides were quantified, and AlamarBlue was used to examine mitochondrial function.

#### 2.2.1. Reduced/Oxidized Glutathione Ratio (GSH/GSSG)

**Figure 2:**
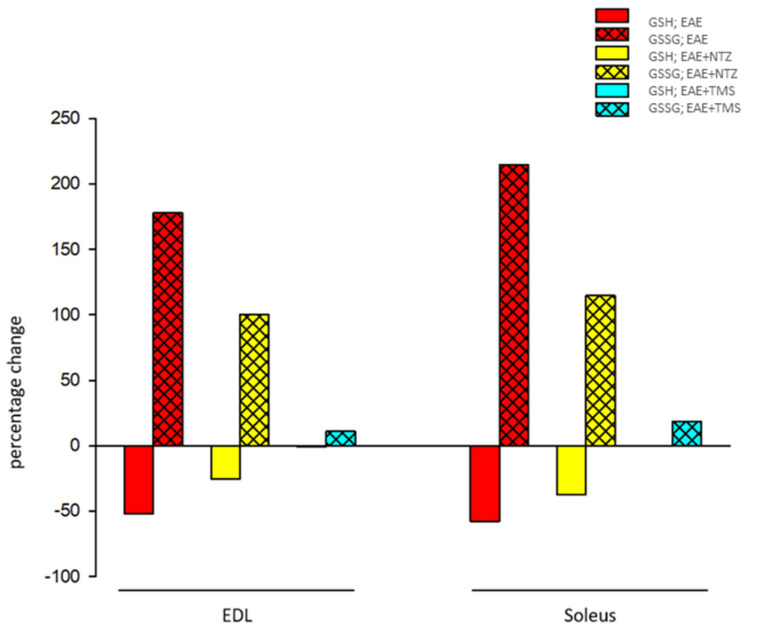
Percentage variations of mean values in the oxidative enzymatic activity parameters for the extensor digitorum longus (EDL) and soleus muscles in the different groups in relation to mean values in control groups.

The GSH/GSSG ratio was significantly lower in the soleus and EDL muscles in the EAE group (87% and 83% decrease, respectively) compared to that of the control and vehicle groups, indicating an increase in oxidative stress (Table 1). Relative to the EAE group, this decrease was significantly less in the treatment groups, although the effect was significantly greater for the EAE + TMS group (Table 1) While in the EAE + NTZ group, the decrease in the ratio with respect to the control was 64% in the EDL muscle and 67% in the soleus, in the EAE + TMS group, it was 13% and 17%, respectively. Given that this decrease could have been produced by decreased GSH, increased GSSG, or by both scenarios simultaneously, we presented these changes as a percentage of variation of mean values in each group in relation to the control groups (Figure 2). As shown, the decrease in the ratio in both muscles was determined by a large increase in GSSC, which was accompanied, to a lesser extent, by a decrease in GSH.

#### 2.2.2. Carbonylated Proteins

There was a significant increase in carbonylated proteins in the EAE group with respect to the control and vehicle groups (by 353% in the EDL and 321% in the soleus). Although the carbonylated proteins remained significantly increased in the EAE + NTZ group compared to the control and vehicle groups (113% for the EDL and 171% for the soleus), they were significantly reduced compared to the EAE group. The increase in the EAE + TMS group with respect to the controls was only 13% for the EDL and 7% for the soleus. Moreover, the reduction in carbonylated proteins was significantly greater in the EAE + TMS treatment group compared to the EAE + NTZ group (Table 2) (Supplementary Figure S2).

#### 2.2.3. Lipoperoxidation Products

The levels of lipoperoxides in the EAE group were significantly increased (by 75% in the EDL and 138% in the soleus) compared to the control group. The increase in the EAE + NTZ group compared to controls was only 23% in the EDL, but surprisingly, it was 150% in the soleus. Regarding the EAE + TMS group, the treatment reduced the levels of lipoperoxides to 5% in the EDL and 4% in the soleus (Table 2).

#### 2.2.4. AlamarBlue

In the EAE group, there was a 56% and 59% decrease, respectively in EDL and soleus compared to the control group. However, at 29% in EDL and 30% in soleus, this decrease was not as profound in the EAE + NTZ group. In the EAE + TMS group, the treatment dampened the decrease in this parameter so that, in comparison with the control, it was only 2% in the EDL and 9% in the soleus. Therefore, its effect was significantly better than the reduction achieved in the EAE + NTZ group (Table 3) (Supplementary Figure S3).

### 2.3. Histology

#### 2.3.1. Muscular Atrophy

The histomorphometric analysis revealed significant changes in the soleus and EDL muscle fiber size parameters between the different groups in terms of the cross-sectional area, diameter (data not shown), and number of muscle fibers/area.

##### Extensor Digitorum Longus Muscle

The histomorphometric results obtained for the EDL muscles are presented in Table 4 and Supplementary Figure S4. Compared with the control group, the cross-sectional area of the muscle fibers in the EAE group was 50%, 49%, and 32% lower for type 1, 2a and 2b fibers, respectively. This atrophy accounted for the increased number of fibers observed per area, which were 52% higher than in the control group. In addition, 26% of the atrophic fibers showed angulated contours and corresponded to type 2b fibers, which explains why the standard deviation of the cross-sectional area was very high.

In the EAE + NTZ group, although the atrophy showed the opposite pattern compared to the EAE group, the type 1 and 2b fibers (but not type 2a fibers) were still significantly smaller than those in the control group. This explains why the number of fibers per area was lower (although not significantly so) than in the EAE group. Moreover, the percentage of angulated fibers in the EAE + NTZ group was significantly reduced (to 7%) with respect to the EAE group.

The histomorphometric results from the EAE + TMS group showed no significant differences in the muscle fiber size with respect to the controls, although there were significant differences compared to the EAE groups and the type 1 and 2b muscle fibers in the EAE + NTZ group. The percentage of angulated fibers was also lower in the EAE + TMS group (as low as 1%) and was significantly different not only from the EAE group but also the EAE + NTZ group.

##### Soleus Muscle

**Figure 3:**
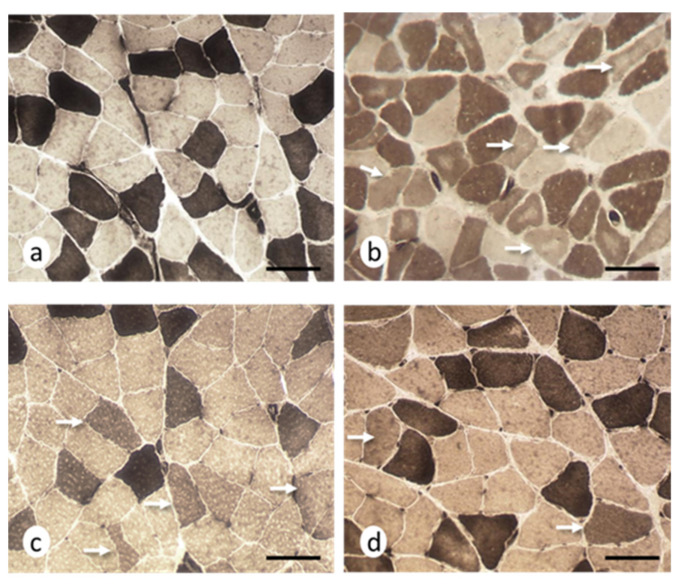
Histochemical evidence of intermediate fibers at day 36. Cross-sections of soleus muscles, (**a**) control group: two histochemical types were observed, type 1 (light) and type 2 (dark) fibers; (**b**) EAE group: fibers with intermediate staining (arrows), some of which showed a central area lacking histochemical activity (core fibers); (**c**) EAE + NTZ group: intermediate fibers (arrows) were observed, some of them with sharp edges; (**d**) EAE + TMS group: the arrows indicate intermediate fibers of an apparently normal size. adenosine triphosphatase (ATPase), pH 9.6. Scale bars = 40 μm.

The histomorphometric results obtained for the soleus muscles are presented in Table 5 and Supplementary Figure S5. The histomorphometric data for the EAE group revealed that both type 1 and type 2 fibers were significantly smaller compared to controls, with a decrease by 28% and 38% for type 1 and 2 fibers, respectively. ATPase (adenosine triphosphatase) staining at pH 9.6 showed the presence of angulated atrophic fibers corresponding to type 2 fibers (Figure 3). This technique also revealed that 8% of the fibers had intermediate staining levels.

In the EAE + NTZ group, both types of muscle fibers were larger than the atrophic muscle fibers found in the EAE group, although there were no significant differences. With respect to the controls, there was 21% atrophy in type 1 fibers and 17% in type 2 fibers, and a reduction to 6% in the intermediate fibers.

The muscle fiber cross-sectional area values in the EAE + TMS group were comparable to those from the controls and were significantly different for type 1 and 2 fibers compared to the EAE group. Only type 1 fibers were significantly larger in size compared to the EAE + NTZ group, with 6% and 5% atrophy with respect to the controls for type 1 and 2 fibers, respectively. In addition, the percentage of intermediate fibers was reduced to 2% in this group.

#### 2.3.2. Neurogenic Lesions in Muscle Fibers

The histology was normal, and the histochemical techniques did not reveal any abnormalities in the control and vehicle groups, and for both the EDL and soleus muscles. In contrast, both muscles presented significant lesions indicative of neurogenic damage (although with different microscopic patterns) in the EAE group; no degenerative or regenerative fibers or inflammatory foci were seen in any of the samples. The data obtained for the treatment groups showed that there was a reduction in the number of muscle fibers showing neurogenic lesions, albeit with certain differences between these two muscles.

##### Extensor Digitorum Longus Muscle

**Figure 4:**
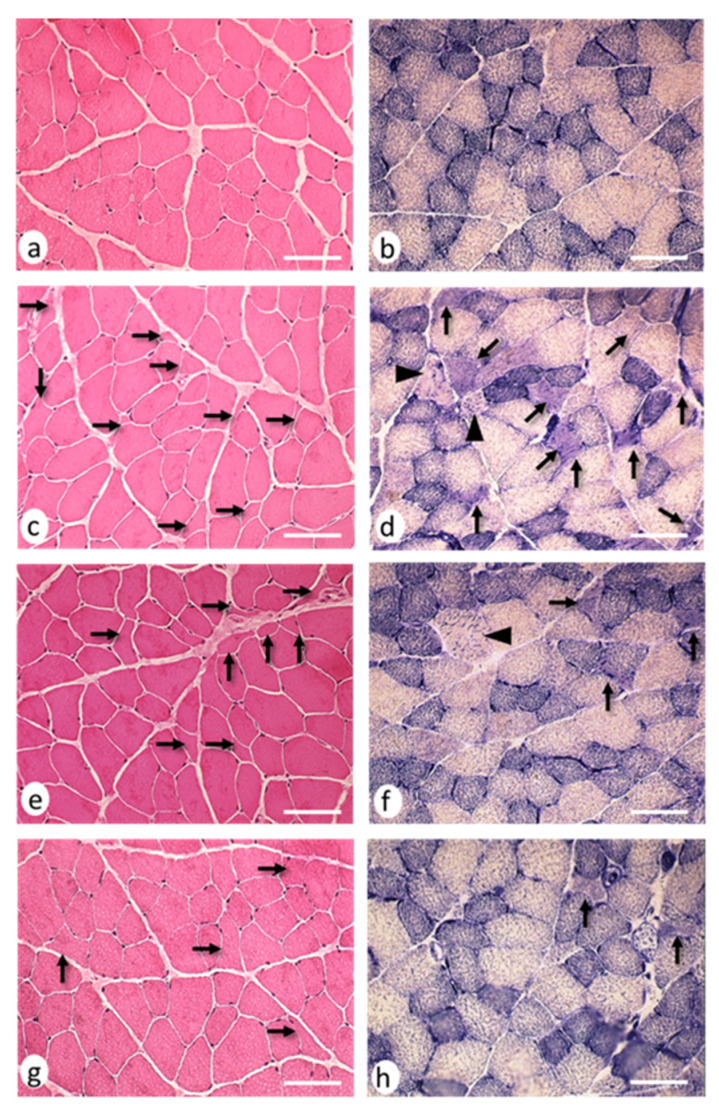
Neurogenic lesions in EDL muscles at day 36. Cross-sections corresponding to (**a**) the control group: muscle fibers showing normal morphology coloration; (**b**) control group: three types of muscle fibers were observed based on their histochemical activity, type 1 (dark), type 2b (light), and type 2a (intermediate); (**c**) EAE group: angulated atrophic fibers (arrows) scattered among apparently normal muscle fibers; (**d**) EAE group: angulated atrophic muscle fibers (arrows) with homogeneous staining and punctiform accumulations of oxidative activity, while other atrophic fibers with rounded contours showed patchy enzymatic activity (moth-eaten fibers; arrow heads); (**e**) EAE + NTZ group: fewer angulated atrophic muscle fibers were observed (arrows); (**f**) EAE + NTZ group: one moth-eaten fiber (arrowhead) and two angulated atrophic muscle fibers; (**g**) EAE + TMS group: the arrows mark several fibers with an angulated profile, although they did not appear atrophic; (**h**) EAE + TMS group: only two atrophic fibers with angular contours were observed (arrows). Images a–g, hematoxylin and eosin staining; images b–h, nicotinamide adenine dinucleotide tetrazolium reductase (NADH-tr) staining. Scale bars = 40 μm.

**Figure 5:**
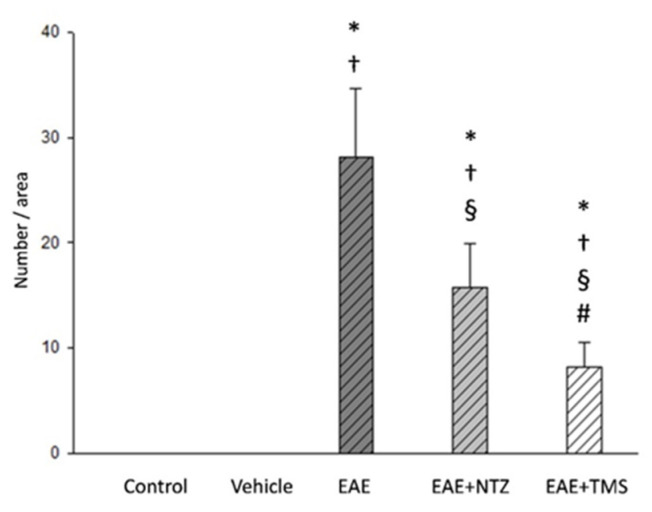
Number of muscle fibers per area with cytoarchitectural lesions in EDL muscle fibers at day 36. Values are means ±SD. There were no lesions detected in control and vehicle groups. * Significantly different (s.d.) from control group; ^†^ s.d. from vehicle group; ^§^ s.d. from EAE group; ^#^ s.d. from EAE + NTZ (*n* = 7 per group).

The H–E (hematoxylin–eosin) staining revealed the presence of small muscle fibers with sharp edges, which were distributed among fibers with an apparently normal morphology (Figure 4a–d). Histochemical stains revealed 28.07 ± 5.61 cytoarchitectural core-multicore myopathy-type lesions and lobulated fibers per area in the EAE group, which significantly reduced (*p* < 0.05) in the EAE + NTZ (15.80 ± 3.10) and EAE + TMS (8.22 ± 2.33) groups (Figure 4e–h) (Figure 5). Despite this decrease in the number of lesions, the percentage of fibers with a given type of lesion did not change in either of the two treatment groups (Supplementary Figure S6).

##### Soleus Muscle

**Figure 6:**
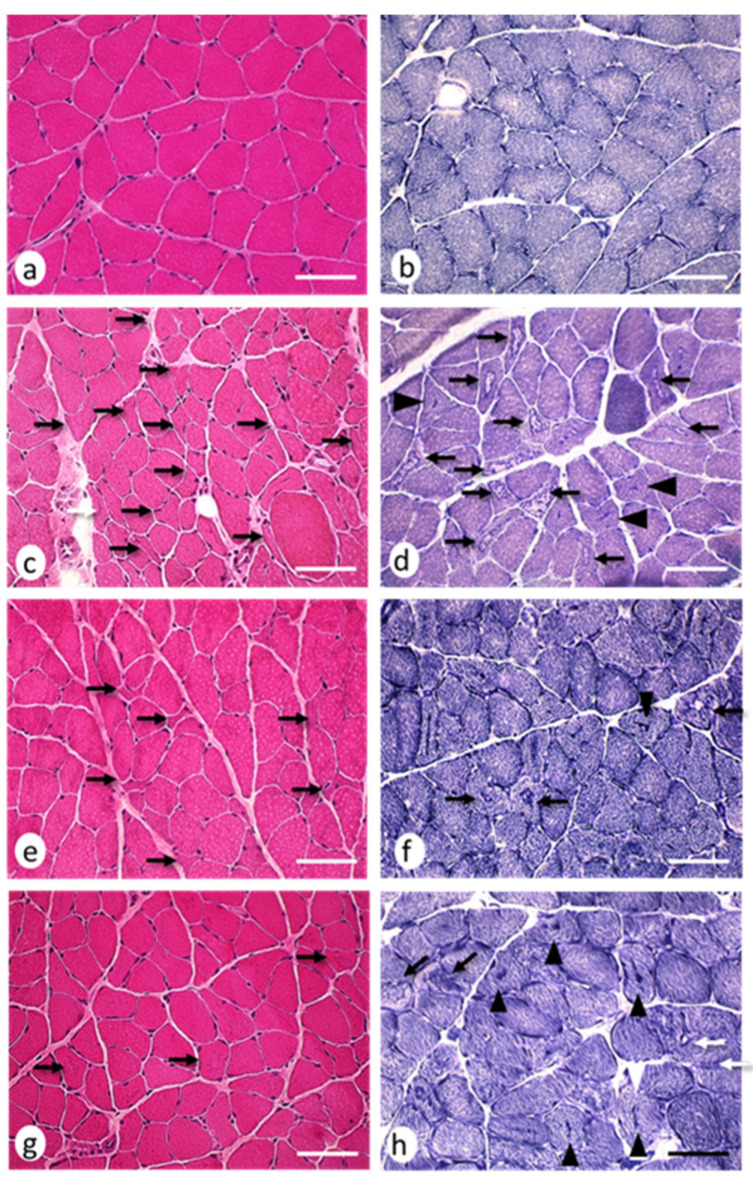
Neurogenic lesions in the soleus muscles at day 36. Cross-sections corresponding to (**a**) control group: muscle fibers showed normal morphology and coloration; (**b**) control group: all muscle fibers showed homogeneous oxidative activity. (**c**) EAE group: muscle fiber atrophy was evident in comparison with the control image, as highlighted by the presence of abundant fibers with an angular profile (arrows); (**d**) EAE group: arrows indicate several atrophic muscle fibers with target fibers (arrows) characterized by an altered pattern of histochemical activity consisting of the presence of concentric areas with different oxidative activity; the arrow heads indicate two fibers with a central spot; (**e**) EAE + NTZ group: fewer angulated atrophic muscle fibers were observed (arrows); (**f**) EAE + NTZ group: target fibers (arrows) and fibers with central spot (arrowhead); (**g**) EAE group + TMS: arrows mark several fibers with a basophilic central area and no angulated atrophic fibers were observed; (**h**) EAE + TMS group: several central spot fibers (arrow heads) and two fibers with a moth-eaten appearance were observed (arrows). Images a–g H&E; images b–h. NADH-tr staining. Scale bars = 40 μm.

**Figure 7:**
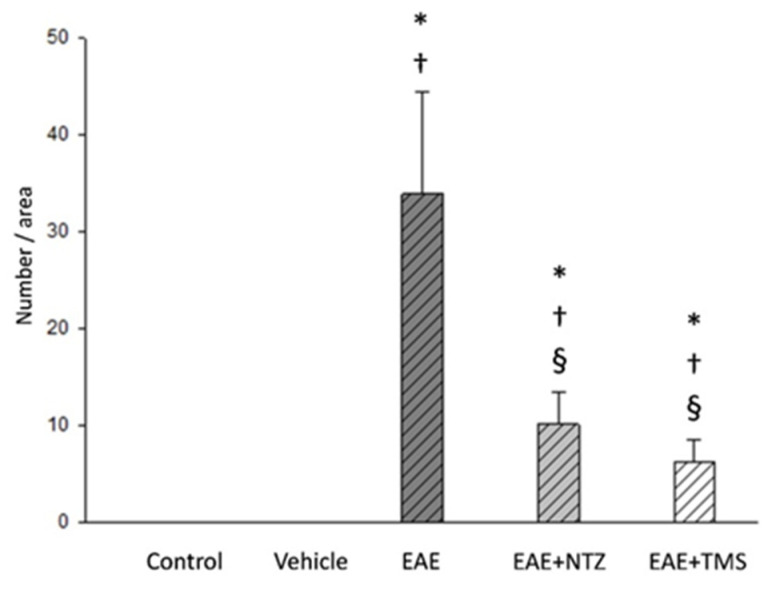
Number of muscle fibers with cytoarchitectural lesions per area in soleus muscle fibers at day 36. Values are means ±SD. There were no lesions detected in control and vehicle groups. * Significantly different (s.d.) from control group; ^†^ s.d. from vehicle group; ^§^ s.d. from EAE group (*n* = 7 per group).

Hematoxylin–eosin staining revealed variability in the size of the muscle fibers, which were smaller and had rounded profiles and, occasionally, angular edges (Figure 6a–d). Moreover, histochemical staining showed the presence of 33.86 ± 10.49 target-targetoid fibers, moth-eaten fibers, and spot fibers per area in the EAE group, which was significantly reduced (*p* < 0.05) in the EAE + NTZ (10.09 ± 3.41) and EAE + TMS (6.23 ± 2.5) groups, although there were no significant differences between the latter two (Figure 6e–h) (Figure 7). The percentage of muscle fibers with a certain type of lesion varied depending on the type of treatment (Supplementary Figure S7).

## 3. Discussion

There were atrophic and cytoarchitectural changes in the muscle fibers in the EAE model indicative of a denervation–reinnervation process associated with increased oxidative stress [13]. The neurogenic involvement of skeletal muscle in the EAE model can be explained as being secondary to axonal damage and demyelination caused by the autoimmune response induced by MOG peptides (in processes involving motor neurons and nerves, as described by other authors). Thus, in mice with EAE induced by MOG peptides, abnormalities in the motor neurons of the lumbo-sacral region [21] as well as axonal damage and demyelination [22] have been described. In this sense, the muscle atrophy and muscle lesions seen in our study seem to indirectly confirm the presence of damage at the level of the central nervous system and reinforce the structural basis, both at the nervous and muscular level, of the motor deficit in the experimental animals, as confirmed in the clinical assessment.

Our results showed that treatment with TMS and NTZ had a beneficial effect on muscle involvement in the EAE model. There was a clinical improvement in functional motor deficits, atrophy was attenuated, neurogenic muscle lesions were reduced, and the level of oxidative stress biomarkers was lower in both treatment groups. Compared to NTZ, the best response was obtained with TMS for all the parameters analyzed. In our opinion, this may reflect differences in the evolution of the pathological processes occurring in the experimental model and in the possible mechanisms of action of both treatments.

While the effect of TMS on skeletal muscle may be related to the activation induced in the central and peripheral nervous systems [23], NTZ works by selectively blocking the alpha chain of the VLA-4 integrin, which inhibits leukocyte migration across the blood–brain barrier [20]. Interestingly, as pointed out by Soellner et al. [24], in the PLP-139–151-induced EAE model, axonal pathology but not the inflammatory myelin pathology increases with disease progression. Based on this finding and the results obtained in this present study, the greater therapeutic effect of TMS compared to NTZ could suggest that, at least in our experimental conditions, the neurogenic muscle lesion component was predominant over the inflammatory one. However, we cannot exclude other mechanisms of action such as the effect of TMS on the central nervous system by secreting anti-inflammatory products and trophic factors that promote regeneration and repair and help restore homeostasis, leading to the stimulation of neuroprotective M2 microglia [25,26]. Thus, all the above could help us understand the mechanisms of recovery from neurogenic muscle atrophy and lesions.

It is clear that MS negatively influences the size of patient muscle fibers as well as the strength and mass of muscles in the lower extremities [10]. Our results confirmed that significant muscle atrophy also occurs in the EAE model and showed that both treatment with NTZ and TMS help to reduce this pathology. However, based on our histomorphometric findings, the antiatrophic effect of TMS was significantly greater than that of NTZ. A recent study found that the application of neuromuscular magnetic stimulation in patients with ALS not only significantly improved their strength, but muscle biopsies also revealed that this treatment reduced atrophy by increasing the size and number of fast-twitch fibers [17]. Moreover, when muscle size was measured by ultrasound analysis, repetitive peripheral magnetic stimulation was shown to prevent atrophy of the rectus femoris muscle of the paretic limb in patients that had suffered an acute cerebrovascular accident [18].

In contrast, an experimental study in rats that were immobilized in a shortened position showed that although magnetic stimulation prevented fibrosis, it did not stop muscle atrophy [19]. TMS depolarizes almost all the spinal motor neurons that innervate the target muscle in healthy individuals [27] and also has a transsynaptic effect [28]. Thus, one could envision that TMS would have a greater therapeutic effect than other agents when there is a ‘denervation’ component or when nerve transmission is affected, and this effect would be reduced when innervation remains intact. Other histological changes, including reduced muscle fiber atrophy and a decrease in microscopic changes indicative of neurogenic damage (intermediate fibers, angulated fibers, and fibers with cytoarchitectural lesions) also suggest that TMS treatment supports increased skeletal muscle recovery compared to NTZ.

In this work, the presence of intermediate muscle fibers—which would be linked to neurogenic injury [13]—in the soleus muscles of rats with EAE was lower in the EAE + TMS group (2.03%) than in the EAE + NTZ group (6.10%). Since the presence of these fibers was related to a decrease in neuromuscular activity [29], a reduction in their number with respect to the EAE group would indicate that treatment with TMS improved neuromuscular activity. However, it has also been pointed out that centrally mediated mechanisms of muscle dysfunction in MS could differentially affect fast-twitch rather than slow-twitch motor units and, therefore, produce different degrees of atrophy in the two fiber groups [7].

The presence of abundant type 2b angular atrophic fibers in this current work was striking and is evidence in support of this possibility in the EAE model. In any case, our results showed that TMS had a positive effect because it significantly reduced the percentage of type 2b angular atrophic fibers seen in the EAE group from 26% to 1%, while the positive effect of NTZ was more limited with a reduction to 7%. Although both TMS and NTZ significantly reduced cytoarchitectural lesions, the former treatment did so in a higher proportion of cases than NTZ. Of note, only TMS reduced the presence of target fibers (20%), while these lesions remained high (79%) with NTZ treatment.

This could be explained by the different mechanisms of action of both these treatments. The presence of target and targetoid fibers are linked to denervation–reinnervation [30], and it seems that they probably correspond to maturational stages of the internal muscle fiber remodeling process [31]. Therefore, the presence of fewer of these fibers in the TMS treatment group reinforces our idea that magnetic stimulation would have accelerated recovery from injuries, probably by stimulating the spinal motor neurons that innervate the target muscle because of a transsynaptic effect. In addition, the higher percentage of fibers with a central spot in the TMS treatment group would indicate recovery in terms of muscle fiber innervation because it represents the final evolutionary phase of target lesions [32]. In contrast, target fibers remained the most common lesion type in the NTZ treatment group, which can be explained by the fact that this drug does not directly affect neurogenic lesions or motor neurons, because its mechanism of action is different to TMS.

Some authors have linked oxidative stress to muscle atrophy, as is the case in diabetic rats [33]. EAE also causes an increase in oxidative stress in both the soleus and EDL muscles [13]. However, it has been pointed out that although denervation induces oxidative stress in skeletal muscle, it does not appear to cause atrophy because antioxidant drug treatments do not prevent the reduction of muscle mass [34]. In our opinion, the increase in oxidative stress in the EAE model might be more strongly related to the changes, caused by denervation, exerted upon the cytological components of muscle fibers. High levels of reactive oxygen species (ROS) promote skeletal muscle contractile dysfunction, resulting in muscle fatigue [35], which in turn has been linked to muscle fiber degeneration and necrosis [36]. However, we did not observe necrotic muscle fibers in the EAE group in this present study nor in our previous work [13].

This lack of correlation between increased ROS and myonecrosis has also been reported in some muscular dystrophy animal models [37]. An increase in ROS in the absence of muscle fiber necrosis can be explained if we consider that superoxide production can occur in different intracellular locations such as mitochondria, sarcoplasmic reticulum, transverse tubes, sarcolemma, cytosol, and myofibrils [35,38]. All these elements are modified or altered during denervation, which leads to cytoarchitectural alterations including target, targetoid, core, and moth-eaten muscle fibers [39], perhaps explaining the increase in oxidative stress in our model. Indeed, mitochondrial respiration significantly reduced, and ROS increased after denervation [40].

The results of our study indicate that another mechanism of action of both these treatments could be the result of reduced oxidative stress. Thus, the antiatrophic effect of TMS could have been favored by its effect on oxidative stress. In a myotoxic-induced muscle degeneration–regeneration model, we found that TMS increased the number and size of regenerative muscle fibers, which was related to a reduction in oxidative stress biomarkers and an increase in nitric oxide (NO) levels [14]. Although we did not analyze NO levels in this present study, it is known that increased NO favors the activation and proliferation of satellite cells [41], which in turn are involved in muscle growth and regeneration processes [42,43]. This is important because the satellite cell population appears to maintain muscle proliferative and differentiation capacity in patients with MS, and the latter can be stimulated by high-intensity training [44]. Therefore, in future work, it would be interesting to analyze the response of satellite cells to treatment with TMS.

Stölting et al. [15] pointed out that magnetic stimulation could effectively support post-traumatic rehabilitation after acute nerve and muscle damage; the results of our study concur with a possible rehabilitative role for TMS. It has been proven that other strategies such as exercise alone were unable to prevent muscle atrophy in the EAE model [45]. Thus, at the muscular level, both atrophy and denervation should be considered in therapeutic strategies to improve functional recovery. However, treating MS with TMS is painless, harmless, and non-invasive [46], and so it could be combined with other treatments to try to design effective therapeutic approaches. This current work verified that TMS significantly reduced target-type lesions and favored recovery from atrophy. Hence, it would be interesting to consider combining its use with exercise in future studies, given that physical activity promotes the reinnervation of muscle fibers [47,48]. Indeed, it has recently been proposed that people with MS should be individually prescribed physical exercise at an early stage, which should be adapted as ‘medicine’ alongside conventional medical treatments [49].

In summary, this work is the first experimental study to demonstrate that TMS can arrest atrophy and reduce the muscle injuries that occur in the EAE model. The data we obtained here, together with those previously published by our group in relation to the central nervous system [2,3,4], provide an experimental rationale for the use of TMS in the treatment of MS.

## 4. Materials and Methods

### 4.1. Experimental Animals and Groups

We used 35, 8-week-old male adult Dark Agouti rats (Janvier Labs, Le Genest-Saint-Isle, France). The animals were kept in cages under controlled temperature conditions at 22 °C with a 12 h light/dark cycle and free access to food (Purina V R, Spain) and water. All the procedures carried out on the animals were approved by the Bioethics Committee at the University of Córdoba (reference number 30 March 2017/053) and were completed in accordance with the Directive of 24 November 1986 (86/609/ECC) approved by the European Communities Council and Royal Decree 53/2013 approved by Head of the Cabinet of Spain (BOE 8 February 2013).

The study included the following groups, each comprising 7 rats:

(i) Control group: comprised of animals without any treatment.
(ii) Vehicle group: EAE was not induced in these rats, but they were inoculated with the vehicle (complete Freund adjuvant) and subsequently maintained without further manipulation until day 36.
(iii) EAE group: EAE was induced on day 1 by subcutaneously injecting a single 100 µL dose of 150 µg of myelin oligodendrocyte glycoprotein ((MOG; fragment 35–55; Sigma, St. Louis, MO, USA) in phosphate-buffered saline (PBS) emulsified 1:1 in a complete Freund adjuvant (Sigma, USA)) into the dorsal base of the tail. To complete the adjuvant, 400 µg of heat-inactivated Mycobacterium tuberculosis (H37Ra, DIFCO, Franklin Lakes, NJ, USA) were also added.
(iv) EAE + NTZ group: EAE was induced, and natalizumab (Tysabri^®^, Biogen Idec, Inc. and Elan Pharmaceuticals, Inc. Cambridge, MA, USA) was intraperitoneally administered in doses of 5 mg/kg body weight on days 15 and 25 [50].
(v) EAE + TMS group: EAE was induced, and animals were treated with TMS. These animals were placed into cylindrical plastic cages designed to keep them immobile. Each coil consisted of 1000 turns of enameled copper wire (7 cm in diameter) contained in plastic boxes (measuring 10.5, 10.5, and 3.5 cm). Two Helmholtz coils generated the electromagnetic fields (Magnetoterapia S.A. de C.V., Mexico D.F., Mexico). The two coils were placed dorsally and ventrally to the head, leaving approximately 6 cm between each coil and the midpoint of the head. The stimulation consisted of an oscillatory magnetic field in the form of a sinusoidal wave with a frequency of 60 Hz and an amplitude of 0.7 mT, applied for two hours in the morning, once a day, 5 days a week (Monday to Friday), for 3 weeks (days 15 to 35), to simulate the application of TMS in clinical practice [51].

### 4.2. Clinical Scoring

Clinical evaluation was carried out by two trained experimenters who were blinded to the experimental conditions. The animals were evaluated on days 1, 14, and 35 according to the following scale [52]: 0 = no signs; 1 = paralysis of the tail; 2 = paralysis of the tail and one limb; 3 = paralysis of the tail and two limbs; 4 = paralysis of the tail and 3 limbs; 5 = paralysis of the tail and 4 limbs.

Both the treatment with NTZ and the application of TMS were implemented from day 15 to day 35. On day 36, the rats were anesthetized and were sacrificed by decapitation. The soleus and EDL muscles were extracted and were subjected to biochemical, histological, and histomorphometric analysis.

### 4.3. Biochemistry

Tissue levels of various oxidative damage and antioxidant markers were quantified in skeletal muscle homogenized in 20 mM Tris-HCl buffer at pH 7.4.

Carbonylated proteins: Protein carbonylation was used as an indicator of protein oxidative stress. Carbonyl content was measured as previously described by Levine et al. [53], using a Shimadzu spectrophotometer (UV-1603; Kyoto, Japan) at a wavelength of 360 nm.

Lipid peroxidation products and glutathione systems: levels of lipid peroxidation products, GSH, and the GSH/ GSSG ratio were measured using Bioxytech LPO-586, GSH-400, and GSH-412 kits, respectively (Oxis International, Portland, OR, USA). The measurements were performed using a UV-1603 spectrophotometer (Shimadzu, Kyoto, Japan).

AlamarBlueTM assay: This redox indicator exhibits both fluorescent and colorimetric changes in response to metabolic activity and can be used to examine mitochondrial function in tissues. AlamarBlue was measured in skeletal muscle tissue preparations using methods adapted from synaptosome studies [54].

Protein estimation: Protein levels were measured using the Bradford method with a B6916 assay kit (Sigma-Aldrich, St. Louis, MO, USA).

### 4.4. Histology

The two soleus and EDL muscles from each rat were frozen in isopentane cooled in liquid nitrogen, and 8 µm serial cuts were obtained at −20 °C using a cryostat. The sections were stained with standard histological and histochemical procedures [55]. Morphology was analyzed after hematoxylin–eosin and modified Gomori trichrome staining. Histochemical techniques employing nicotinamide reduced nicotinamide adenine dinucleotide dehydrogenase-tetrazolium reductase (NADH-tr) and ATPase at pH 9.6 were used to identify the muscle fiber types.

Five areas of each muscle (EDL and soleus) were photographed at a ×400 magnification using a Nikon Eclipse E1000 microscope (Nikon, Tokyo, Japan) equipped with a Sony Exwave HAD camera, covering a total area of 263,200 µm^2^ in each case. We performed the histomorphometric analysis with the Image-Pro Plus 6.0 image analysis program (Media Cybernetics, Bethesda, MA, USA), analyzing the following parameters in each overall area, in each muscle from each animal: (1) cross-sectional area (µm2) and smallest muscle fiber diameter (µm), (2) number of fibers per area, (3) percentage of angulated atrophic fibers, and (4) percentage of different types of muscle fibers. Morphometric analysis was performed in the soleus muscle with ATPase histochemical staining at pH 9.4 to highlight type 1 and type 2 fibers, and in the EDL muscle with NADH-tr staining to distinguish type 1, 2a and 2b fiber types. The cytoarchitectural lesions seen in the muscle fibers were also quantified and were expressed as a percentage where appropriate.

### 4.5. Statistical Analysis

The two homologous muscles from each animal were considered as the same unit for the statistical analysis of the biochemical and histomorphometric parameters. The results were expressed as the mean ± the standard deviation (SD). For statistical analysis of histomorphometric parameters, one-way ANOVAs followed by Newman–Keuls tests were performed to detect inter-group differences. Holm–Sidak tests were performed for normal data and ANOVA on ranks followed by Dunn tests were performed for non-normal data. Statistical analysis of the histomorphometric variables was carried out using Sigma Stat 3.1 software (Systat Software Inc., Bengaluru, India). Analysis of the biochemical parameters, weight, and scores was performed using SPSS software (version 17.7, SPSS Inc., Chicago, IL, USA) A one-way ANOVA was carried out to evaluate data variation. The level of statistical significance was set at *p* < 0.05.

## Figures and Tables

**Table 1:** Antioxidant enzymatic activities in EDL and soleus muscles of the different groups.

	GSH/GSSG		GSH (nMol/mg Protein × 10)		GSSG (nMol/mg Protein × 10)	
	EDL	Soleus	EDL	Soleus	EDL	Soleus
**Control**	3.71 ± 0.32	3.83 ± 0.22	0.0098 ± 0.00015	0.0102 ± 0.00078	0.0027 ± 0.00034	0.0027 ± 0.00041
**Vehicle**	3.68 ± 0.26	3.99 ± 0.35	0.0098 ± 0.00026	0.0104 ± 0.00093	0.0028 ± 0.00036	0.0026 ± 0.00051
**EAE**	0.63 ±0.11 *^,†^	0.51 ±0.07 *^,†^	0.0047 ± 0.00017 *^,†^	0.0043 ± 0.00031 *^,†^	0.0075 ± 0.00041 *^,†^	0.0085 ± 0.00059 *^,†^
**EAE + NTZ**	1.35 ± 0.14 *^,†,§^	1.25 ± 0.25 *^,†,§^	0.0073 ± 0.00037 *^,†,§^	0.0064 ± 0.00037 *^,†,§^	0.0054 ± 0.00042 *^,†,§^	0.0058 ± 0.00042 *^,†,§^
**EAE + TMS**	3.23 ± 0.48 ^§,#^	3.18 ± 0.21 *^,†,§,#^	0.0097 ± 0.00055 ^§,#^	0.0102 ± 0.00086 ^§,#^	0.0030 ± 0.00031 ^§,#^	0.0032 ± 0.00034 ^§,#^

All values are expressed as mean ± SD. * *p* < 0.05 versus control group; ^†^ *p* < 0.05 versus vehicle group; ^§^ *p* < 0.05 versus EAE group; ^#^ *p* < 0.05 versus EAE + NTZ group.

**Table 2:** Values of carbonylated proteins (CP) and lipid peroxidation products (LPO) in skeletal muscle.

	CP (nMol/mg Protein)		LPO (nMol/mg Protein)	
	EDL	Soleus	EDL	Soleus
**Control**	0.015 ± 0.002	0.014 ± 0.03	0.125 ± 0.012	0.121 ± 0.007
**Vehicle**	0.016 ± 0.001	0.015 ±0.002	0.117 ± 0.010	0.111 ± 0.015
**EAE**	0.068 ± 0.003 *^,†^	0.059 ±0.004 *^,†^	0.219 ± 0.034 *^,†^	0.288 ± 0.018 *^,†^
**EAE + NTZ**	0.032 ± 0.003 *^,†,§^	0.038 ± 0.002 *^,†,§^	0.154 ± 0.006	0.303 ± 0.031 *^,†^
**EAE + TMS**	0.017 ± 0.002 ^§,#^	0.013 ± 0.002 ^§,#^	0.131 ± 0.012 ^§^	0.126 ± 0.014 ^§,#^

All values are expressed as mean ± SD. * *p* < 0.05 vs. control group; ^†^ *p* < 0.05 vs. vehicle group; ^§^ *p* < 0.05 versus EAE group; ^#^ *p* < 0.05 vs. EAE + NTZ group.

**Table 3:** Values of AlamarBlue (AB) fluorescence.

AB Fluorescence (Arbritary Units)		
	EDL	Soleus
**Control**	225.20 ± 14.97	240.60 ± 23.00
**Vehicle**	213.20 ± 22.68	213.20 ± 22.69
**EAE**	98.20 ± 9.44 *^,†^	98.20 ± 9.45 *^,†^
**EAE + NTZ**	161.00 ± 22.12 *^,†,§^	168.33 ± 11.14 *^,†,§^
**EAE + TMS**	219.80 ± 14.16 ^§,#^	218.67 ± 17.62 ^§,#^

All values are expressed as mean ± SD. * *p* < 0.05 versus control group; ^†^ *p* < 0.05 versus vehicle group; ^§^ *p* < 0.05 versus EAE group; ^#^ *p* < 0.05 versus EAE + NTZ group.

**Table 4:** Histomorphometric parameters of EDL muscle.

	Cross-Sectional Area (µm^2^)			Fiber Number/Area	% Angulated Atrophic Fibers
	Type 1 Fiber	Type 2a Fiber	Type 2b Fiber		
**Control**	926.64 ± 84.07	1175.61 ± 178.27	2139.04 ± 191.42	28.33 ± 2.23	0
**Vehicle**	836.39 ± 101.81	1142.05 ± 128.09	2065.31 ±210.03	29.28 ± 3.53	0
**EAE**	460.96 ± 71.50 *^,†^	605.12 ± 85.3 *^,†^	1462.07 ± 402.08 *	45.01 ± 6.3 *^,†^	26.03 ± 4.30 *^,†^
**EAE + NTZ**	640.53 ± 58.53 *^,†,§^	1096.35 ± 140.22 ^§^	1504.12 ± 242.21 *^,†^	37.62 ± 4.34 *^,†^	7.16 ± 2.18 *^,†,§^
**EAE + TMS**	827.03 ± 89.67 ^§,#^	1200.23 ± 164.69 ^§^	1952.91 ± 196.62 ^§^^,#^	29.75 ±2,41 ^§,#^	0.92 ± 1.80 *^,†,§,#^

All values are expressed as mean ± SD. * *p* < 0.05 versus control group; ^†^ *p* < 0.05 versus vehicle group; ^§^ *p* < 0.05 versus EAE group; ^#^ *p* < 0.05 versus EAE + NTZ group.

**Table 5:** Histomorphometric parameters of soleus muscles.

	Cross-Sectional Area (µm^2^)			Fiber Number/Area	% Angulated Atrophic Fiber	% Fiber Type 1	% Fiber Type 2	% Intermediate Fiber Type
	Fiber Type 1	Fiber Type 2	Intermediate Fiber Type					
**Control**	1845.28 ± 166.80	1717.23 ± 244.85	0	41.11 ± 3.79	0	91.11 ± 2.31	8.89 ± 1.23	0
**Vehicle**	1689.23 ± 147.16	1629.24 ± 194.54	0	39.29 ± 4.21	0	89.41 ± 2.33	10.58 ± 2.20	0
**EAE**	1322.90 ± 132. 71 *^,†^	1032. 49 ± 132.83 *^,†^	1125. 02 ± 174.33 *^,†^	87.31± 7.80 *^,†^	26.79 ± 3.22 *^,†^	60.15 ± 3.94 *^,†^	31.64 ± 2.78 *^,†^	8.21 ± 1.12 *^,†^
**EAE + NTZ**	1450.31 ± 173. 02	1422.26 ± 198.03 ^§^	1486.69 ± 204.74 *^,†^	54.22 ± 4.31 *^,^^†,§^	20.29 ±3.71 *^,^^†^	68.79 ± 2.68 *^,†,§^	26.01 ± 3.29 *^,†^	6.10 ± 0.40 *^,†,§^
**EAE + TMS**	1737.35 ± 152.04 ^§^	1634.09 ± 234.41 ^§^	1696.27 ± 237. 39 *^,†,^^§^	41.71 ± 4.32 ^§,#^	9.43 ± 4.12 *^,†,§,#^	81.36 ± 2.12 *^,†,§,#^	14.61 ± 2.71 *^,§,#^	2. 03 ± 0.32 *^,†,§,#^

All values are expressed as mean ± SD. * *p* < 0.05 versus control group; ^†^ *p* < 0.05 versus vehicle group; ^§^ *p* < 0.05 versus EAE group; ^#^ *p* < 0.05 versus EAE + NTZ group.

## Data Availability

Not applicable.

## Supplementary Materials

The following are available online at https://www.mdpi.com/article/10.3390/ijms22168589/s1.
