# Supplementary material for: Transcranial Magnetic Stimulation Improves Muscle Involvement in Experimental Autoimmune Encephalomyelitis
Source: Int J Mol Sci. 2021 Aug 10;22(16):8589. doi: 10.3390/ijms22168589 (PMC8395284; doi:10.3390/ijms22168589)
Supplement: Supplementary file 1 [file ijms-22-08589-s001.zip › ijms-1292111-supplementary.pdf]

SUPPLEMENTARY MATERIAL

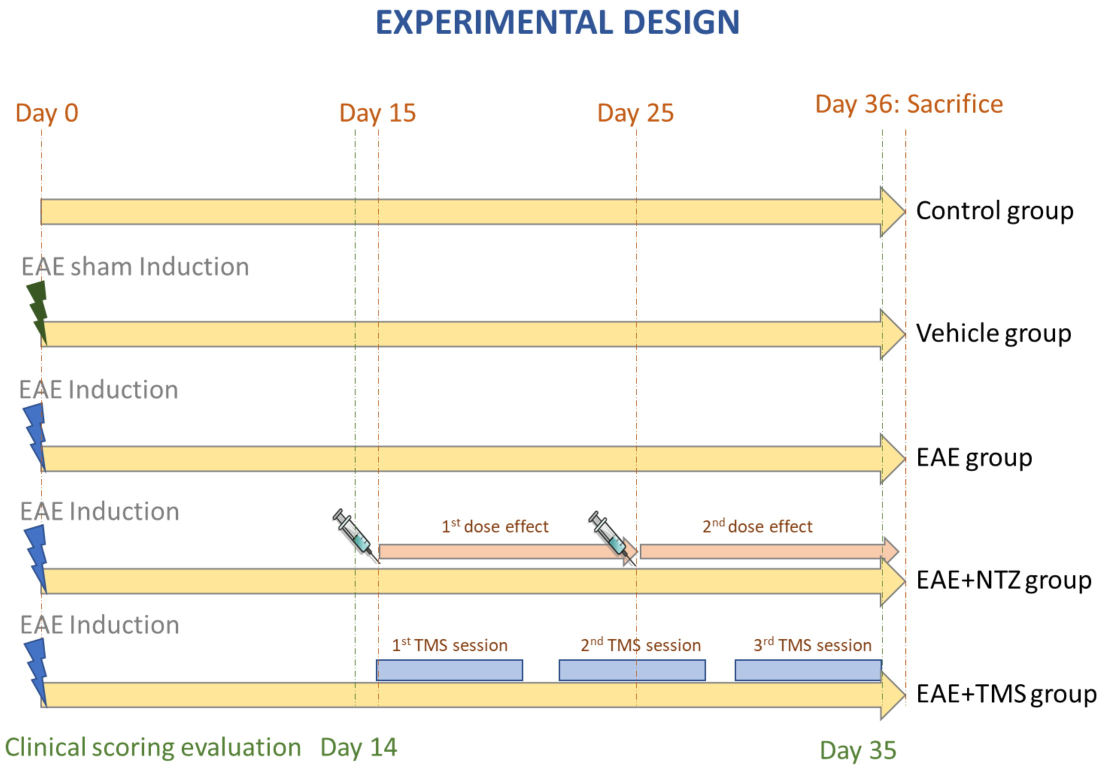

Supplementary Figure S1. Schematic diagram of experimental design.

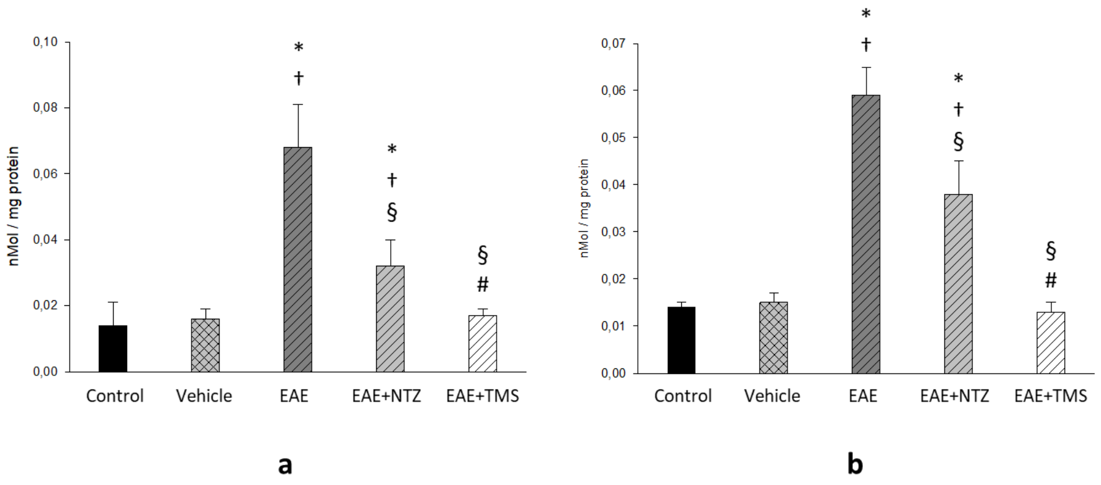

Supplementary Figure S2. Carbonylated proteins. (a) EDL muscle. (b) Soleus muscle. Value are means ±SD. *Significantly different (s.d.) from control group; †s.d. from vehicle group; § s.d. from EAE group; #s.d. from EAE+NTZ group.

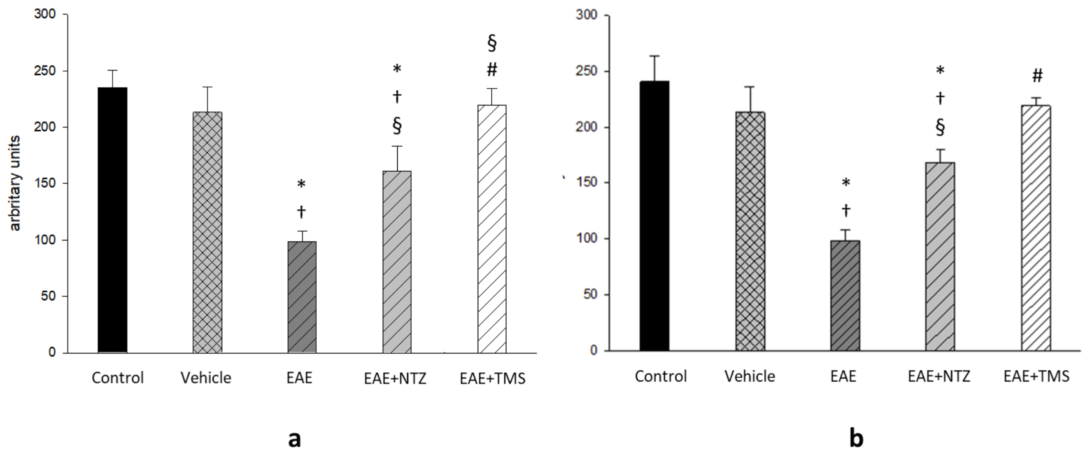

Supplementary Figure S3. AlamarBlue. (a) EDL muscle. (b) Soleus muscle. Value are means ±SD. *Significantly different (s.d.) from control group; †s.d. from vehicle group; § s.d. from EAE group; #s.d. from EAE+NTZ group.

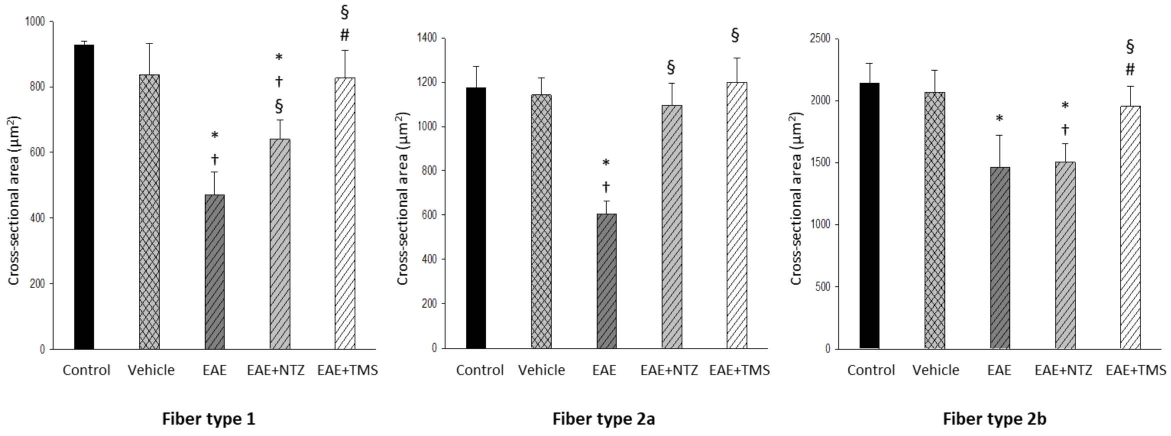

Supplementary Figure S4. EDL muscle. Cross-sectional area of skeletal muscle fibers. Value are means ±SD. *Significantly different (s.d.) from control group; †s.d. from vehicle group; § s.d. from EAE group; #s.d. from EAE+NTZ group.

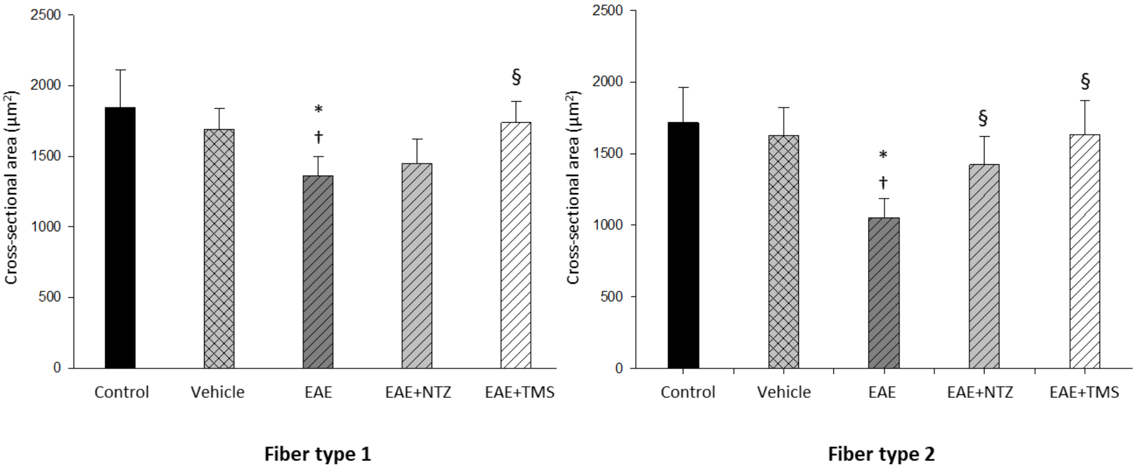

Supplementary Figure S5. Soleus muscle. Cross-sectional area of skeletal muscle fibers. Value are means ±SD. *Significantly different (s.d.) from control group; †s.d. from vehicle group; § s.d. from EAE group; #s.d. from EAE+NTZ group.

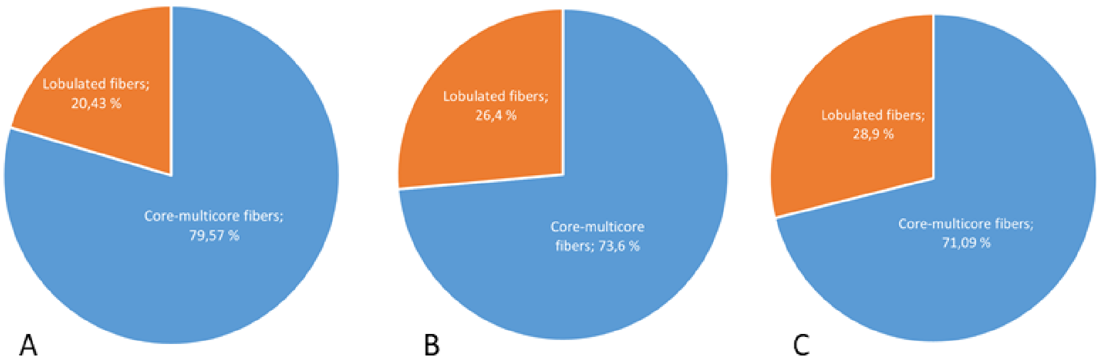

Supplementary Figure S6. Percentage of cytoarchitectural type of lesions according to the group at day 36 in EDL muscles. (A) EAE group, (B) EAE+NTZ, (C) EAE+TMS.

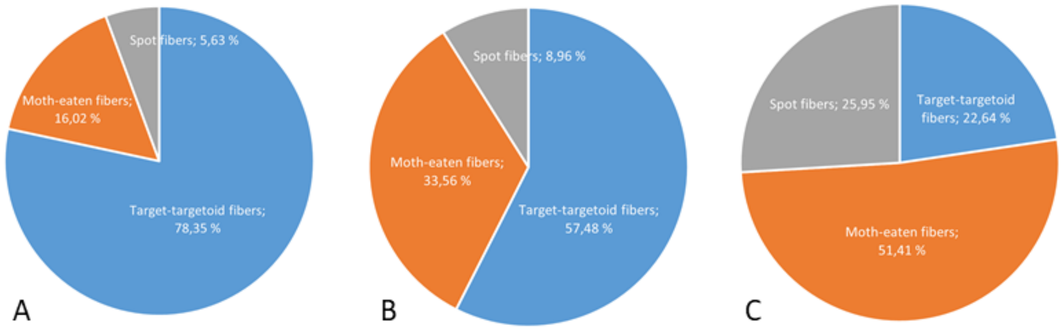

Supplementary Figure S7. Percentage of cytoarchitectural type of lesions according to the group at day 36 in soleus muscles. (A) EAE group, (B) EAE+NTZ, (C) EAE+TMS.
